# DMSO reduces the cytotoxicity of anticancer ruthenium complex KP1019 in yeast

**DOI:** 10.17912/micropub.biology.000436

**Published:** 2021-08-04

**Authors:** Jonathan Davis, Anne Cetto, Mary Campbell, Seth Scoggins, Laura Stultz, Pamela Hanson

**Affiliations:** 1 Department of Biology, Furman University, Greenville, SC 29613, USA; 2 Department of Chemistry, Birmingham-Southern College, Birmingham, AL 35254, USA

## Abstract

Low solubility in aqueous solutions is a significant limitation of the otherwise promising anticancer ruthenium complex KP1019. In laboratory studies, this challenge is often overcome by using DMSO to help drive the drug into solution. Since DMSO was previously shown to alter the bioactivity of platinum-based chemotherapeutics, here we examine DMSO’s effects on KP1019. Using *Saccharomyces cerevisiae* as a model organism, we apply multiple measures of growth inhibition to demonstrate that DMSO reduces the drug’s toxicity. This reduction in bioactivity correlates with spectrophotometric changes consistent with DMSO-dependent increases in the stability of the KP1019 pro-drug. The impact of DMSO on the biology and chemistry of KP1019 suggests this solvent should not be used in studies of this and similar anticancer ruthenium complexes.

**Figure 1. DMSO decreases the cytotoxicity and increases the spectrophotometric stability of KP1019:**
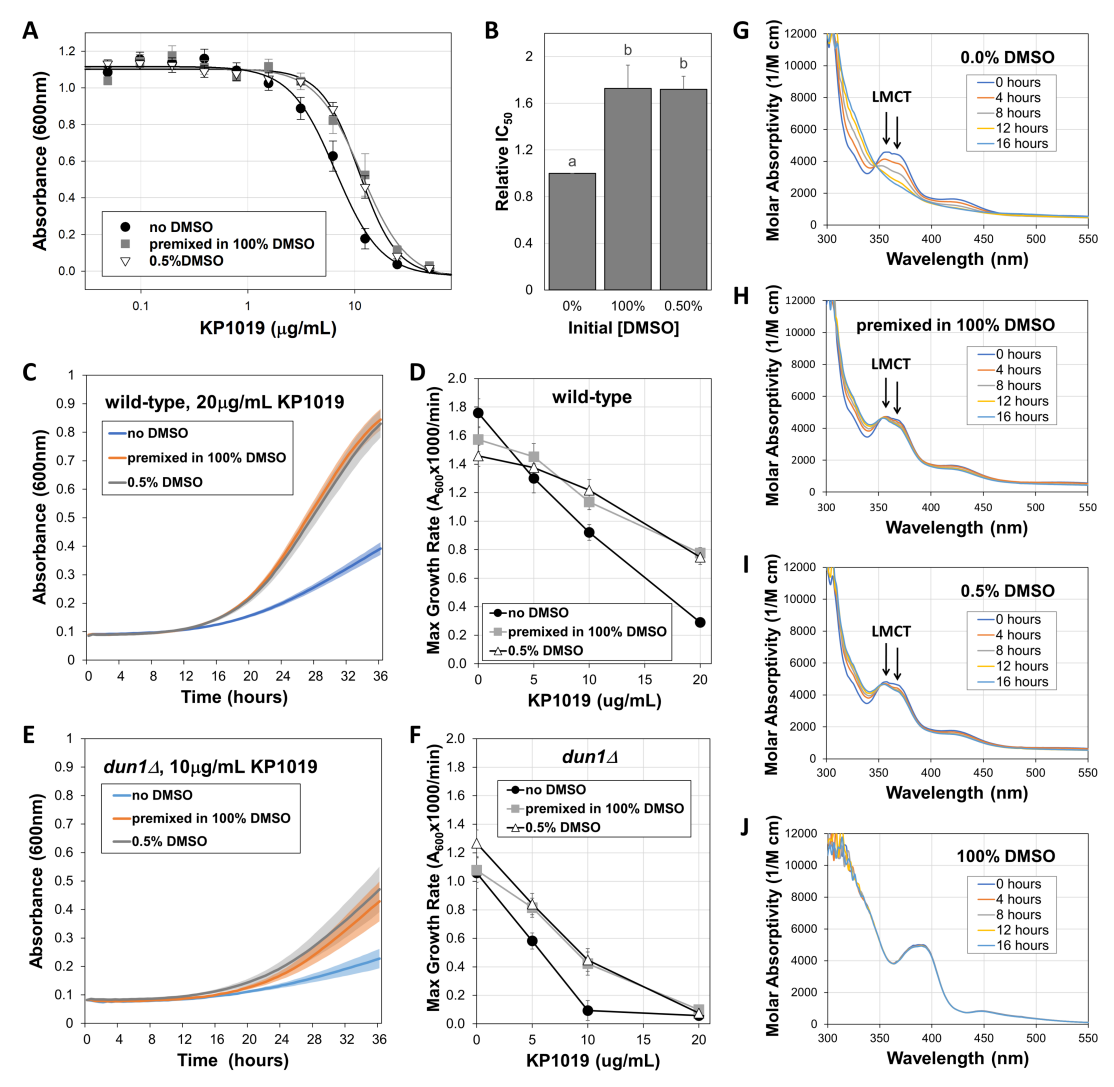
**A)** Yeast were grown in varying KP1019 concentrations with or without DMSO for 20 hours prior to measuring growth as absorbance at 600 nm. Data shown represent the average and standard error for 6 biological replicates fit with 4-parameter logistic curves. **B)** Growth inhibition data from **A** were used to calculate “relative IC_50_” values wherein IC_50_ values for experimental samples were divided by the IC_50_ of the no DMSO control; bars represent average and standard error (n=6). Letters above error bars represent significantly different groups (p<0.05) as determined by paired student t-tests. **C)** Growth of wild-type yeast was measured over time in the presence of 20µg/mL KP1019 solubilized three different ways. Lines indicate average absorbance at 600nm and shading surrounding lines corresponds to standard error of six biological replicates. **D)** The maximum growth rates from **C** and analogous trials featuring other drug concentrations were plotted as a function of drug concentration. Graph depicts the mean and standard error of six biological replicates. **E and F)** Growth curves and dose response experiments were conducted on *dun1∆* yeast, using protocols similar to those used for **C** and **D**, except **E** represents data gathered at 10µg/mL KP1019 (average ± S.E., n=6). **G-J)** UV-vis spectra at four-hour intervals for 0.25mM KP1019 in phosphate buffer **(G)**, buffer with DMSO **(H-I)**, or pure DMSO **(J)**. Data correspond to one representative trial per condition.

## Description

In an early stage dose-escalation clinical trial, anticancer ruthenium complex indazolium *trans*-[tetrachlorobis(1*H*-indazole)ruthenate(III)] (also known as KP1019), stabilized disease progression in five of six evaluable patients (Hartinger *et al.* 2008). However, KP1019’s low solubility prevented identification of the maximum tolerated dose. In laboratory studies, the challenge of KP1019’s poor solubility is often overcome by dissolving the drug in dimethyl sulfoxide (DMSO) before diluting it into the growth medium or buffer being used in a particular assay (Bergamo *et al*. 2009; Singh *et al.* 2014; Golla *et al.* 2017; Śpiewak *et al.* 2019). Though many studies control for the biological impacts of DMSO itself by including a vehicle only control, the impacts of DMSO on KP1019’s chemistry and bioactivity have not been directly tested. Notably, a previous study showed that the activities of the platinum drugs cisplatin and carboplatin are reduced by 87 to 98% respectively when solubilized with DMSO (Hall *et al*. 2014). Given this finding and ruthenium’s high affinity for sulfur containing ligands, we hypothesized that DMSO would also affect KP1019.

To determine whether DMSO increases or decreases KP1019 bioactivity, dose-response growth inhibition assays were conducted with wild-type *Saccharomyces cerevisiae*, which is an appropriate model for studying KP1019, because both budding yeast and cancer cells experience similar effects from the drug. For example, KP1019 damages DNA and induces oxidative stress in both cancer and yeast cells (Kapitza *et al.* 2005; Golla *et al*. 2017; Stultz *et al*. 2020). Additionally, multidrug resistant cancer and yeast cell lines both remain sensitive to KP1019 treatment (Heffeter *et al.* 2005; Stevens *et al.* 2013). Since prior studies dissolved KP1019 in DMSO before dilution in aqueous solutions, we compared this approach to dissolving KP1019 directly in yeast media. As seen in Figure 1A, premixing KP1019 with pure DMSO shifts the dose response curve to the right, significantly increasing growth at multiple KP1019 concentrations. Consistent with a recent study, which reported the IC_50_ for KP1019 in the absence of DMSO as 6.8µg/mL (Stultz *et al.* 2020), here we found a similar value of 6.7±0.8µg/mL (mean ± S.E., n=6) for the same, widely used wild-type strain BY4742. More importantly, initially dissolving the drug in DMSO prior to dilution in yeast media significantly increased the IC_50_ to 11.4±1.8µg/mL.

To determine whether this loss of efficacy was dependent on initially dissolving KP1019 in pure DMSO, we also conducted the growth inhibition assay with drug that was dissolved directly in media containing a final concentration of 0.5% DMSO. This approach also reduced KP1019’s efficacy, raising the IC_50_ to 11.2±0.9µg/mL, which is statistically comparable to the value obtained by first dissolving the drug in pure DMSO (p>0.05). These changes in efficacy are summarized in Figure 1B in which the IC_50_’s for each condition are normalized to the no DMSO control for each trial. These data illustrate that inclusion of DMSO increases KP1019’s IC_50_ by greater than 50%, representing a significant loss of bioactivity. This reduction in effective dose has potential to be clinically significant, as a roughly one-third drop in dose or dose intensity for other chemotherapies has been shown to worsen patient outcomes (Bonadonna *et al.* 1995; Budman *et al.* 1998)

As a complement to the end-point readings used for IC_50 _analyses, growth curve assays were conducted in the presence of select concentrations of KP1019 solubilized with or without DMSO. As seen in Figure 1C, when the drug was dissolved in 100% DMSO prior to dilution in yeast media, or when KP1019 is dissolved in media containing 0.5% DMSO, yeast growth is substantially less inhibited with the average maximum growth rate increasing more than 2.5-fold from 0.29 ± 0.02 for the no DMSO control to 0.78 ± 0.04 and 0.75 ± 0.05 A_600_x1000/min (mean ± S.E., n=6), respectively. These results verify that DMSO reduces the toxicity of KP1019 in yeast. Likewise, other KP1019 concentrations resulted in dose-dependent decreases in the maximum growth rate of wild-type yeast (Figure 1D), and multivariate regression analyses verified that this growth inhibition was reduced in the presence of DMSO (p<0.05). Notably, there was not a statistically significant difference between the two approaches for including DMSO in the assay (p>0.05).

Analogous to observations in wild-type yeast, DMSO also reduced the efficacy of KP1019 against yeast lacking the kinase Dun1 (Figures 1E and 1F), which is essential for the yeast DNA damage response (Zhou and Elledge 1993). Whereas Figures 1D and 1F verify that *dun1∆* yeast have a modest growth defect relative to wild-type strains even in the absence of drugs (Marek *et al*. 2013), Figures 1C-F verify previous observations that *dun1∆* strains are hypersensitive to KP1019 (Singh *et al.* 2014; Stultz *et al.* 2020). For example, at 10µg/mL KP1019 in the absence of DMSO, the maximum growth rate of wild-type yeast is 0.92 ± 0.06 A_600_x1000/min (mean ± S.E.) (Figure 1D). Under these same conditions, the *dun1∆* strain grows at roughly one tenth the rate of the wild-type, reaching a maximum of 0.09 ± 0.07 A_600_x1000/min (Figure 1F). In the *dun1∆* mutant, the effects of DMSO are clear. For example, at 10µg/mL KP1019, inclusion of DMSO resulted in a more than four-fold increase in average growth rate, reaching 0.42 ± 0.08 and 0.45 ± 0.08 A_600_x1000/min when pre-mixed with 100% DMSO or dissolved in media containing 0.5% DMSO, respectively. However, these growth rates are still much lower than the wild-type controls. This sustained KP1019 sensitivity of the *dun1∆* yeast suggests that even DMSO-impaired KP1019 can damage DNA. However, future studies will be required to establish whether DMSO interferes with KP1019’s other cellular impacts, such as proteotoxicity and oxidative stress (Kapitza *et al.* 2005; Stultz *et al.* 2020).

To determine whether DMSO’s effects on KP1019 bioactivity correlate with changes to the drug’s chemical properties, UV-vis spectra were analyzed. As seen in Figure 1G, the spectrum for KP1019 dissolved in buffer alone initially shows characteristic peaks at 357 nm and 421 nm, which are indicative of ligand to metal charge transfer (LMCT) (Kratz *et al.* 1994). These peaks gradually decrease over time as the drug’s chloride ligands are exchanged for water (Küng *et al.* 2001). When initially dissolved in DMSO then diluted in buffer (Figure 1H) or when dissolved in buffer already containing 0.5% DMSO (Figure 1I), the LMCT bands are largely maintained over the 16-hour timeframe of the experiment. As a control, we also tested KP1019 in pure DMSO for the duration of the experiment. As seen in Figure 1J, this condition shifts the LMCT peaks to the right (390 nm and 448 nm), and the spectrum does not change over the timeframe of the experiment. Altogether, these data may indicate that DMSO increases the stability of KP1019 in solution. This increased stability suggests that DMSO may interfere with KP1019’s activation by reduction, which is required for the metal to bind and damage biomolecules like DNA (Antonarakis and Emadi 2010). Though our data do not conclusively rule out formation of a highly stable complex that contains DMSO, the similarity of the initial spectra for aqueous solutions with and without DMSO (Figures 1G-I), make ruthenium binding directly to DMSO an unlikely explanation in this case, as such binding would be anticipated to alter the LMCT bands. Instead DMSO may have a strong solvent interaction with KP1019, which could prevent the reduction required for activation and binding to cellular components (Antonarakis and Emadi 2010).

In light of KP1019’s limited aqueous solubility, the sodium salt analog KP1339 was developed (Trondl *et al*. 2014). This drug contains the same active metal complex but has a sodium counterion rather than indazolium, making KP1339 far more soluble than KP1019. Even still, some studies of KP1339 have used DMSO as a vehicle (Flocke *et al.* 2016; Bakewell *et al.*. 2020), and we predict this solvent would have similar effects on KP1339 as we report here for KP1019. Given DMSO’s impact on KP1019, caution should be used when comparing studies that do and do not dissolve the drug in DMSO, and when possible, future research on ruthenium complexes should avoid using DMSO as a vehicle.

## Methods

*Drug Synthesis*

KP1019 (CHEBI ID: 77760) was synthesized as described previously (Stevens *et al*. 2013).

*Determination of IC_50_ and Growth Curve Analysis*

KP1019’s ability to inhibit yeast growth was quantified in terms of an IC_50_ as described previously with the following modifications (Stevens *et al.* 2013). KP1019 was dissolved in SDC yeast media, dissolved in 100% DMSO and then diluted in SDC with a final DMSO concentration of 0.5%, or dissolved in SDC containing 0.5% DMSO. To maintain a consistent vehicle concentration, drug solutions were two-fold serially diluted in media with or without 0.5% DMSO. Overnight *Saccharomyces cerevisiae* cultures were normalized to A_600_ 0.1, then diluted 20-fold more in SDC with or without 0.5% DMSO as appropriate. An equal volume of cell suspension was added to the serially diluted KP1019. Relative concentrations of KP1019 were estimated with a pre-incubation scan at 360 nm, and trials where different experimental groups (DMSO vs. no DMSO) were more than 10% away from the mean were excluded from further analyses. Plates were incubated at 30°C for 20 hours before end-point absorbance (600nm) was measured. A similar approach was used for growth curve assays, except mid-log phase cultures were used to reduce the duration of lag phase, microtitre plate lids were pre-treated to prevent accumulation of condensation (Brewster 2003), plates were incubated at room temperature, and absorbance was measured at 30-minute intervals for 36 hours. Maximum growth rates were determined automatically by Biotek Gen5 software, which calculated the maximum slope by evaluating 5 points post lag phase.

*UV-vis spectrophotometry*

Absorbance spectra of solutions containing a final concentration of 0.25mM KP1019 were studied under four different conditions: dissolved directly in 0.5 M sodium phosphate buffer (pH 7.2) without DMSO, pre-dissolved in 100% DMSO then diluted to 0.5% DMSO in phosphate buffer, dissolved directly in phosphate buffer containing 0.5% DMSO, and dissolved in 100% DMSO only. Data was collected at 25°C every hour for 19 hours, and absorbance was converted to molar absorptivity by dividing by the concentration of KP1019. For clarity, representative scans at 0, 4, 8, 12, and 16 hours were graphed.

## Reagents

The yeast strains used in this study were BY4742 (MATα *his3Δ1 leu2Δ0 lys2Δ0 ura3Δ0*) (Brachmann *et al.*. 1998) and a commercially available, isogenic *dun1::kanMX* strain (Winzeler *et al.* 1999).

## References

[R1] Antonarakis ES, Emadi A (2010). Ruthenium-based chemotherapeutics: are they ready for prime time?. Cancer Chemother Pharmacol.

[R2] Bakewell S, Conde I, Fallah Y, McCoy M, Jin L, Shajahan-Haq AN (2020). Inhibition of DNA Repair Pathways and Induction of ROS Are Potential Mechanisms of Action of the Small Molecule Inhibitor BOLD-100 in Breast Cancer.. Cancers (Basel).

[R3] Bergamo A, Masi A, Jakupec MA, Keppler BK, Sava G (2009). Inhibitory Effects of the Ruthenium Complex KP1019 in Models of Mammary Cancer Cell Migration and Invasion.. Met Based Drugs.

[R4] Bonadonna G, Valagussa P, Moliterni A, Zambetti M, Brambilla C (1995). Adjuvant cyclophosphamide, methotrexate, and fluorouracil in node-positive breast cancer: the results of 20 years of follow-up.. N Engl J Med.

[R5] Brachmann CB, Davies A, Cost GJ, Caputo E, Li J, Hieter P, Boeke JD (1998). Designer deletion strains derived from Saccharomyces cerevisiae S288C: a useful set of strains and plasmids for PCR-mediated gene disruption and other applications.. Yeast.

[R6] Brewster JD (2003). A simple micro-growth assay for enumerating bacteria.. J Microbiol Methods.

[R7] Budman DR, Berry DA, Cirrincione CT, Henderson IC, Wood WC, Weiss RB, Ferree CR, Muss HB, Green MR, Norton L, Frei E 3rd (1998). Dose and dose intensity as determinants of outcome in the adjuvant treatment of breast cancer. The Cancer and Leukemia Group B.. J Natl Cancer Inst.

[R8] Flocke LS, Trondl R, Jakupec MA, Keppler BK (2016). Molecular mode of action of NKP-1339 - a clinically investigated ruthenium-based drug - involves ER- and ROS-related effects in colon carcinoma cell lines.. Invest New Drugs.

[R9] Golla U, Swagatika S, Chauhan S, Tomar RS (2017). A systematic assessment of chemical, genetic, and epigenetic factors influencing the activity of anticancer drug KP1019 (FFC14A).. Oncotarget.

[R10] Hall MD, Telma KA, Chang KE, Lee TD, Madigan JP, Lloyd JR, Goldlust IS, Hoeschele JD, Gottesman MM (2014). Say no to DMSO: dimethylsulfoxide inactivates cisplatin, carboplatin, and other platinum complexes.. Cancer Res.

[R11] Hartinger CG, Jakupec MA, Zorbas-Seifried S, Groessl M, Egger A, Berger W, Zorbas H, Dyson PJ, Keppler BK (2008). KP1019, a new redox-active anticancer agent--preclinical development and results of a clinical phase I study in tumor patients.. Chem Biodivers.

[R12] Heffeter P, Pongratz M, Steiner E, Chiba P, Jakupec MA, Elbling L, Marian B, Körner W, Sevelda F, Micksche M, Keppler BK, Berger W (2004). Intrinsic and acquired forms of resistance against the anticancer ruthenium compound KP1019 [indazolium trans-[tetrachlorobis(1H-indazole)ruthenate (III)] (FFC14A).. J Pharmacol Exp Ther.

[R13] Kapitza S, Jakupec MA, Uhl M, Keppler BK, Marian B (2005). The heterocyclic ruthenium(III) complex KP1019 (FFC14A) causes DNA damage and oxidative stress in colorectal tumor cells.. Cancer Lett.

[R14] Kratz F, Hartmann M, Keppler B, Messori L (1994). The binding properties of two antitumor ruthenium(III) complexes to apotransferrin.. J Biol Chem.

[R15] Küng A, Pieper T, Wissiack R, Rosenberg E, Keppler BK (2001). Hydrolysis of the tumor-inhibiting ruthenium(III) complexes HIm trans-[RuCl4(im)2] and HInd trans-[RuCl4(ind)2] investigated by means of HPCE and HPLC-MS.. J Biol Inorg Chem.

[R16] Marek A, Korona R (2013). Restricted pleiotropy facilitates mutational erosion of major life-history traits.. Evolution.

[R17] Singh V, Azad GK, Mandal P, Reddy MA, Tomar RS (2014). Anti-cancer drug KP1019 modulates epigenetics and induces DNA damage response in Saccharomyces cerevisiae.. FEBS Lett.

[R18] Śpiewak K, Świątek S, Jachimska B, Brindell M. 2019. Induction of transferrin aggregation by indazolium [tetrachlorobis (1 <i>H</i>-indazole) ruthenate (iii)](KP1019) and its biological function. New J Chem. 43: 11296-11306.

[R19] Stevens SK, Strehle AP, Miller RL, Gammons SH, Hoffman KJ, McCarty JT, Miller ME, Stultz LK, Hanson PK (2012). The anticancer ruthenium complex KP1019 induces DNA damage, leading to cell cycle delay and cell death in Saccharomyces cerevisiae.. Mol Pharmacol.

[R20] Stultz LK, Hunsucker A, Middleton S, Grovenstein E, O'Leary J, Blatt E, Miller M, Mobley J, Hanson PK (2020). Proteomic analysis of the S. cerevisiae response to the anticancer ruthenium complex KP1019.. Metallomics.

[R21] Trondl R, Heffeter P, Kowol CR, Jakupec MA, Berger W, Keppler BK. 2014. NKP-1339, the first ruthenium-based anticancer drug on the edge to clinical application. Chem Sci. 5: 2925-2932.

[R22] Winzeler EA, Shoemaker DD, Astromoff A, Liang H, Anderson K, Andre B, Bangham R, Benito R, Boeke JD, Bussey H, Chu AM, Connelly C, Davis K, Dietrich F, Dow SW, El Bakkoury M, Foury F, Friend SH, Gentalen E, Giaever G, Hegemann JH, Jones T, Laub M, Liao H, Liebundguth N, Lockhart DJ, Lucau-Danila A, Lussier M, M'Rabet N, Menard P, Mittmann M, Pai C, Rebischung C, Revuelta JL, Riles L, Roberts CJ, Ross-MacDonald P, Scherens B, Snyder M, Sookhai-Mahadeo S, Storms RK, Véronneau S, Voet M, Volckaert G, Ward TR, Wysocki R, Yen GS, Yu K, Zimmermann K, Philippsen P, Johnston M, Davis RW (1999). Functional characterization of the S. cerevisiae genome by gene deletion and parallel analysis.. Science.

[R23] Zhou Z, Elledge SJ (1993). DUN1 encodes a protein kinase that controls the DNA damage response in yeast.. Cell.

